# Patient preferences for key organizational features of primary cardiovascular care in Quebec: a discrete choice experiment

**DOI:** 10.1186/s12875-025-02810-4

**Published:** 2025-04-10

**Authors:** Claudio Del Grande, Janusz Kaczorowski, Marie-Pascale Pomey

**Affiliations:** 1 Health Innovation and Evaluation Hub, University of Montreal Hospital Research Centre, Montreal, QC Canada; 2 Department of Family Medicine and Emergency Medicine, Faculty of Medicine, Université de Montréal, Montreal, QC Canada; 3 Department of Management, Evaluation and Health Policy, School of Public Health, Université de Montréal, Montreal, QC Canada

**Keywords:** Patient preferences, Primary care, Family practice, Cardiovascular disease, Chronic disease, Organization of care, Accessibility, Continuity, Discrete choice experiment

## Abstract

**Background:**

Cardiovascular diseases and their risk factors are leading causes of morbidity and mortality worldwide, and are among the top reasons for primary care visits. Little is known about patient preferences for primary care in the context of chronic conditions. This study aimed to investigate the effect of key organizational features identified by patients and providers on patients’ choice of a preferred primary care practice to receive cardiovascular care.

**Methods:**

A discrete choice experiment survey was completed by a weighted online sample of 501 Quebec residents having or being at risk of cardiovascular disease. Respondents completed one of two blocks of nine choice sets by indicating, among three hypothetical primary care practice alternatives in each choice set, their preferred and second-most preferred options. Alternatives were differentiated on the basis of five key attributes identified as priorities in an earlier Delphi study: listening to and respecting care preferences; providing personalized information; 24-to- 48-h accessibility in the event of a problem; continuity of care; and up-to-date clinical skills. Each attribute could be assigned a best, moderate, or worst level. Choices were analyzed using generalized multinomial logit modeling. Marginal effects and choice probabilities for policy-relevant scenarios were estimated.

**Results:**

All five attributes significantly influenced choices of primary care practice. The marginal effects of worst attribute levels were of much greater magnitude than those of best levels for all attributes. Improving short-term accessibility from worst to moderate level had the largest average incremental effect on the probability of patients choosing a practice. Best continuity of care was more valued by older patients and those in poorer general health, but had nonsignificant impact unless it was coupled with enhanced short-term accessibility.

**Conclusions:**

A balanced approach across the key organizational features covered seems more advantageous for primary care practices than focusing solely on achieving excellence in any single attribute. The interactions between patient preferences for short-term accessibility and continuity of care should be taken into account when planning and implementing organizational change in primary care. Whether these preferences are generalizable to other jurisdictions and subsets of primary care patients deserves further exploration.

**Supplementary Information:**

The online version contains supplementary material available at 10.1186/s12875-025-02810-4.

## Background

Cardiovascular diseases (CVD) and their risk factors, such as hypertension, dyslipidemia, and diabetes, are among the leading causes of morbidity and mortality globally [1, 2]. Virtually everyone is at risk of requiring cardiovascular healthcare during their lifetime, as approximately 90% of normotensive middle-aged and elderly individuals are expected to develop high blood pressure, the leading risk factor for CVD [3]. In several jurisdictions, primary care settings are responsible for the routine prevention and management of chronic conditions, including CVD. Hypertension and diabetes are among the most common reasons for visiting a primary care provider in many parts of the world [4]. In a high-COVID region of Canada, these also remained among the top five reasons for primary care visits during the COVID- 19 pandemic [5].

Primary care, by its very definition, encompasses the fundamental principles of accessibility, continuity, coordination, comprehensiveness, technical quality, and efficiency in delivering acute, chronic, and preventive care within the framework of family and community [6–8]. Due to its multidimensional and complex nature, primary care delivery has often remained a “black box” for policy-makers [6, 9]. Several observational studies have found associations between organizational characteristics of primary care practices and their performance on specific dimensions of care [10–13]. However, research has not identified an overall “winning” formula, leaving clinical settings with the difficult task of managing trade-offs between competing dimensions of care with little empirical guidance [14]. Understanding the importance patients place on organizational features and dimensions of primary care can contribute to priority-setting and structure care delivery by being more responsive to their preferences and needs.

Among different methods available to investigate patient preferences, discrete choice experiments (DCE) have become increasingly popular to inform a wide range of health services and policy questions in the context of scarce resources and limited real-world data [15–17]. A DCE is a quantitative research method that assumes that the value placed on a good or service is based on its attributes. In a DCE survey, participants are presented with a series of hypothetical options for the good or service under consideration from which they must choose (e.g., health interventions, treatment options, health services or policies). These options are defined using a number of attributes, which can in turn take on different values. By analyzing the choices made, researchers can identify which attributes are most important to participants, and how much weight is given to different levels of each attribute [18].

Several DCE studies have focused on patient preferences for primary care. A systematic review of such studies by Kleij et al. [19] found no consistent pattern in their preference choices, but estimates from DCE studies are known to be influenced by many study design features, including the study population, choice context, set of attributes and range of attribute levels examined, experimental design, and model specification [18, 20]. Careful tailoring of these factors with the targeted knowledge mobilization context is thus essential for DCE results to be relevant to end-users. An updated systematic review by Lim et al. [21] covering all DCE studies on patient preferences for primary care published until 2021 reported a paucity of evidence for chronic conditions. This prompted the authors to recommend that future DCE studies focus more on these conditions, given the central role of primary care in meeting the preventive care and ongoing management needs of patients with long-term conditions. To our knowledge, no DCE has been conducted to explore patient preferences regarding the organization of primary cardiovascular care despite the considerable burden and widespread prevalence of CVD. To fill out this knowledge gap, we set out to conduct a DCE with cardiovascular patients in Quebec to investigate the effect of priority organizational features on the choice of a primary care practice. Our study also aimed to estimate the expected choice probabilities of different policy-relevant scenarios of primary care practice.

## Methods

Our DCE was administered through an online survey. We followed best-practice guidelines for the design, analysis and report of DCE studies [22–25]. Ethical approval was obtained from the University of Montreal Hospital Research Centre’s research ethics committee (project number 17.305).

### Study setting

Quebec is Canada’s largest province in land area and the second most populous, with around 9 million residents. Each Canadian province and territory has a health insurance plan which provides universal coverage for medical services that is funded with assistance from federal cash and tax transfers. While several primary care practice models coexist in Quebec, including traditional solo clinics and local community health centers, family medicine groups are the main organizational model [26]. A family medicine group is generally composed of 6 to 12 family physicians who work together and in close collaboration with registered nurses and allied healthcare professionals (such as psychologists and social workers) to care for patients enrolled in the practice. This practice model is supported by a government funding and professional support program [27]. Affiliated patients remain free to consult other primary care providers, including walk-in clinics. However, access to other providers remains limited and few patients, if any, have the opportunity to choose their primary care setting, settling for the one they have, when they have one at all. According to Quebec’s statistics institute, over one in four residents were not affiliated with a primary care setting in 2023. Family physicians are free to accept or reject new patients, and receive a premium for registering orphan patients from a centralized waiting list managed by the government. Nevertheless, many enrolled patients turn to emergency departments for their acute care needs due to lack of timely access to their usual primary care source.

### Attribute and level development

The attributes included in our DCE were identified from a previous study conducted using the Delphi technique [28]. The methods and results of this study have been published elsewhere [29]. Briefly, 36 panelists (20 patients and 16 providers) were recruited from urban, suburban, and remote primary care practices in Quebec. These panelists were asked to formulate, in free-text fields, key features to optimally organize primary cardiovascular care for patients, and then to assess their importance by rating and ranking them in iterative rounds of questionnaires interspersed with controlled feedback. Out of an initial set of 41 items, six proved to be highly prioritized by both patients and providers at the end of the structured communication process. These top items, which were consistent with broader research on patient priorities for the organization of primary care and chronic illness care [19, 30–33], formed the basis for the attributes in our DCE. However, to reduce the complexity of our choice tasks, two priority items related to relational and informational continuity were merged into a single continuity of care attribute encompassing both aspects. The key factor in interpreting this combined attribute relates to ensuring that there is a link (fluidity) between the patients’ visits so that they do not have to repeat their story and retrace their care trajectory at every encounter. Thus, our DCE study covered five key organizational features of primary care: listening to and respecting patient care preferences; providing personalized information to patients; 24-to- 48-h accessibility in the event of a problem; continuity of care; and up-to-date clinical skills. All but one attribute definition mentioned “professionals” in the plural to reflect the move toward team-based care in primary care settings that has been underway for the past twenty years in many jurisdictions, including Canada [34]. Because Canada has a universal, publicly funded healthcare system in which most primary care services are free at the point of use, a cost attribute was not included.

Three levels representing a worst (low), moderate, and best (high) amount were set for each attribute to account for potential nonlinear effects (see Table 1). Primary care accessibility in Quebec and Canada is relatively poor [35–37], and the levels used in other DCE studies for this attribute (e.g., ‘no waiting time’, ‘1 day’, ‘2 days’, ‘5 days’ until appointment [19]) seemed unrealistic with the estimated average waiting time of 24 days in Quebec [38]. Thus, our levels were set around how frequently a patient would be able to reach a clinic professional within a 24-to- 48-h delay in the event of a problem. This formulation also resonated with policy targets set by the Quebec government [39]. The levels for up-to-date clinical skills were calibrated around the five-year reference period for the continuing professional development cycle of family physicians in Quebec and Canada. A plausible range was determined by consulting with five practicing family physicians.

**Table 1:** Attributes and levels in the experiment

Attributes	Descriptions	Levels
Listening to and respecting patient care preferences	How much professionals listen to you in order to respect your requests, choices, preferences and motivation in care decisions	A little; ^*^moderate; a lot
Providing personalized information	How much detailed information about your own health status (check-ups, personal risks, etc.) is shared by professionals during consultations	A little; ^*^moderate; a lot
24-to- 48-h accessibility	How often you can reach a clinic professional within 24–48 h if you have a problem, either on site, by phone or by teleconsultation (e.g., video conferencing)	^*^Rarely; about every other time; always or almost
Continuity of care	How much the consulted professionals know you and have easy access to all your health information to ensure there is a link between your visits	A little; ^*^moderate; a lot
Up-to-date clinical skills	The speed with which the clinic's professionals keep up with new ways to better care for you	Every 8–10 years; ^*^every 4–5 years; every 1–2 years

^*^Reference level

### Experimental design

Our DCE followed a best-best design [40–42], in which participants were asked to choose their most preferred (first-best) and second-most preferred (second-best) options sequentially in choice sets of three unlabeled, hypothetical primary care practices. No opt-out option was provided. A near-orthogonal fractional factorial design was constructed to allow estimation of main attribute effects and prespecified interaction effects between 24-to- 48-h accessibility and continuity of care, due to reported tensions and synergies between these two attributes [10, 14, 43–48]. The experimental design was optimized in SAS 9.4 (SAS Institute Inc.) based on the relative D-efficiency criterion, with priors set to zero [49]. Dominant and dominated alternatives were prevented from occurring within choice sets [49], as they provide no relevant information on how respondents make trade-offs between attributes [23]. An alternative is dominant when all its attribute levels are at least as good or better than all the attribute levels of another alternative, which becomes dominated. Optimal designs with 18, 27, and 36 choice sets were examined. Due to negligible improvement in D-efficiency with larger designs, we opted for the 18-set design (83.9% D-efficiency). To further reduce respondent burden [50], the design was divided into two blocks of nine uncorrelated choice sets [49], to which participants were randomly assigned.

### Survey design and target population

**Fig. 1:**
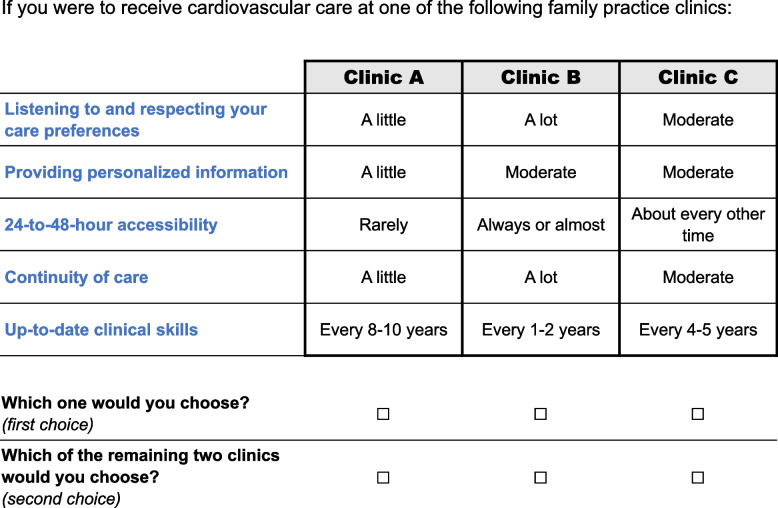
The example choice set. Legend: The example choice set in the survey, which deliberately contained a dominant (Clinic B) and a dominated (Clinic A) option

The online survey, available in French and English, had four sections. Appendix A1 in Additional file 1 presents the English version. The first section contained the information and consent form. The second section assessed participants’ basic profile and eligibility. Eligible respondents were Quebec residents aged 35 and over, with CVD or with a metabolic CVD risk factor (hypertension, dyslipidemia, or diabetes, excluding gestational diabetes). Profile and eligibility questions were taken from the Canadian Community Health Survey (CCHS) [51], an annual cross-sectional survey from Canada’s national statistical office. The third section of the survey pertained to the DCE. Respondents were instructed to answer each choice set as if they had to choose where to receive their primary cardiovascular care in real life. At any time, they could hover or click on an attribute to bring up its detailed description (see Table 1). All respondents completed an example choice set (Fig. 1) to familiarize themselves with the task before being randomized to their block of choice sets. This example deliberately contained a dominant and a dominated alternative, and answers were not included in the analyses. After the DCE, we assessed perceived task difficulty by using a standard user metric known as the Single Ease Question (SEQ) [52], a 7-point rating scale from 1- “very difficult” to 7- “very easy”.

The fourth survey section contained additional sociodemographic and health-related variables. These were selected based on their typicality in subgroup analyses and their relevance according to the panelists that had taken part in our prior Delphi study [29] (see Appendix A2 in Additional file 1). They included: main occupation, level of education, household income, general health, assessment of chronic comorbidities, severity of the cardiovascular health condition, importance attached to health, health literacy level, and readiness to change lifestyle. The first five covariables were measured by using the corresponding CCHS questions [51]. Health literacy was measured by using the BRIEF Health Literacy Screening Tool [53], a four-question self-report instrument validated in English and French [54, 55]. Readiness to change diet, physical activity level, tobacco, and alcohol consumption were measured separately by using 1-to- 10 Readiness Rulers [56, 57]. Such rulers with verbal anchors have been extensively used and adapted to support motivational interviewing in primary care settings [58, 59].

Three quality control questions were incorporated throughout the survey. Participants responding incorrectly to one of these questions were warned to be more careful and to fully read the rest of the questions carefully. Those failing two attention checks were prevented from continuing the survey and their responses were discarded. A soft launch of the survey was made with the first 33 respondents to test for problems with survey design and functioning. No problem was found and the data from these participants were included in the final dataset.

### Data collection

We worked with Leger Opinion [60] (LEO), the largest proprietary panel in Canada, to coordinate the online survey programming, participant recruitment and data collection. The LEO panel includes more than 400 000 members across Canada and the United States mainly recruited randomly by traditional and mobile phone methodologies to increase representativeness. More than 70% of LEO panel members are recruited based on random selection using LEO’s call center. The other panelists are recruited through invitation and affiliate programs, social media, partner campaigns, word of mouth and offline recruitment based on various criteria (no river sampling). All LEO panel members are double opted-in and have gone through screening processes to prevent duplicate accounts and fraud. Several quality checks are in place, including mandatory profile update every six months and removal of sleepers; identification of cheaters or speeders and removal of repeat offenders; phone number verifications; and blocking of suspicious email addresses, domain names or IP addresses. When a survey is sent out, LEO uses proprietary software that draws on Canadian census data to generate samples that are more representative of the population, by factoring in a response rate based on age, gender, and region. Our survey was only sent to individuals identified in the LEO database as residing in Quebec and aged 35 and over, based on information in their verified profile and their IP address (access via VPN is blocked). Participants were not offered additional incentive for completing our survey outside of LEO’s built-in reward system, which includes earning chances to participate in contests to win prizes and receiving points that can be redeemed for online money transfers, credit or gift cards. We aimed to obtain 500 completed surveys. There is no gold standard approach to sample size calculation in a DCE, but this number largely exceeded the minimum sample size required to estimate main effects (n = 56) and all two-way interactions (n = 167) according to Johnson and Orme’s rule of thumb [61] (see Appendix A3 in Additional file 1 for calculations). However, we were also interested in estimating covariable effects to explore whether patient preferences varied according to some of their characteristics, which require larger sample sizes [18].

### Statistical analysis

Attribute levels were dummy coded, with the reference categories selected to reflect an average primary care practice in Quebec. Thus, the moderate level was set as the reference for all attributes, except for 24-to- 48-h accessibility where the worst level was chosen. Rank-ordered logit (ROL) models were used to analyze the choice data to take advantage of the full ranking of alternatives provided by our best-best design. As different individuals can be expected to make different decisions in the same choice situations, taking preference heterogeneity into account reduces bias in the parameters estimated by the choice model [24]. To allow for flexible substitution patterns and account for unexplained heterogeneity in individual preferences (that is, variability not attributable to any patient characteristic examined), we used generalized multinomial logit (G-MNL) modeling [62] on the “exploded” ROL data, as proposed by Lancsar et al. [41], in what we call generalized rank-ordered logit (G-ROL) models.

Three progressive G-ROL models were estimated with standard errors clustered at the respondent level, using 1 000 draws for the simulation with the Broyden-Fletcher-Goldfarb-Shanno optimization method [63]. The first model (G-ROL1) included only main effects modelled as normally distributed random coefficients. The second model (G-ROL2) added the interaction terms between accessibility and continuity as fixed parameters, since no heterogeneity was found on three out of the four interaction coefficients. The final model (G-ROL3) accounted for explained preference heterogeneity (that is, variability attributable to observed patient characteristics) by adding, as fixed effects, significant covariable interactions from the second and last survey sections, including CVD status (with CVD or at risk of CVD) (see Appendix A4 in Additional file 1 for details). Based on the final model (G-ROL3), marginal effects and choice probabilities of selected primary care practice scenarios were calculated.

Sampling weights were applied in all our analyses to fit our respondents to the Quebec population according to the latest CCHS weighted dataset available at the time (2017–2018). Data weighting can reduce the accuracy of results, which is a concern when combined with a small sample size. However, we felt that the cost in reduced accuracy would be acceptable with 500 respondents. Weighting factors were calibrated based on participants’ sex, age group (in 10-year increments), education level (high school or less, postsecondary), and CVD status. For example, a sampling weight was given to all female respondents in our sample that were aged between 35–44 years, had a high school education level or less, and were at risk of CVD (without having CVD), so that their proportion in our sample would be equivalent to that in the CCHS weighted dataset for the Quebec population (weight = % in population divided by % in sample). The same procedure was repeated for all respondent subgroups. We excluded respondents who selected the dominated alternative in the example choice set as their first or second choice (n = 34), because we suspected that they might not have sufficiently understood the attribute levels or paid attention to the choice tasks. Sensitivity analyses were performed on our final model (G-ROL3) by including these respondents and by using unweighted data.

Statistical analyses were conducted in Stata 18.0 (StataCorp LLC, College Station, TX). Statistical significance was set at *p* < 0.05. G-ROL models and choice predictions were estimated using the gmnl and gmnlpred commands, respectively [64].

## Results

Data collection took place from November 5 to November 12, 2021 and was stopped after 501 completed questionnaires were obtained. At that moment, 2 656 survey invitations had been sent to LEO panel members: 1 371 individuals (51.6%) had not yet opened the invitation message; 667 (25.1%) had opened the invitation but had not completed the consent form; 50 (1.9%) had refused to take part in the study; 14 (0.5%) had agreed but were ineligible based on the screening questions; 51 (1.9%) had not completed the DCE portion of the survey; and 2 respondents had been excluded for failing two attention checks. The characteristics of participants with complete choice data as well as the subset that was included in the final model are presented in Table 2, with and without sampling weights. Around 20% of our sample had CVD, and a third reported having comorbid mental and physical health conditions. Compared to the Quebec population aged ≥ 35 with a cardiovascular health condition, our unweighted sample overrepresented the younger age groups and underrepresented the oldest as well as those with a lower education level. Most participants completed the survey within a plausible time frame of between 11 and 19 min. The interquartile range of SEQ scores obtained (3–5) sat right in the middle of the scale, indicating that the choice task was neither found to be very easy nor very difficult by most respondents.

**Table 2:** Characteristics of respondents

Characteristics	Total *N* = 501		In final model^†^ *N* = 466	
	**n (%) unweighted**	**(%) weighted**	**n (%) unweighted**	**(%) weighted**
Age range, in years 35–44 45–54 55–64 65–74 ≥ 75	55 (11.0) 99 (19.8) 167 (33.3) 145 (28.9) 35 (7.0)	(6.1) (15.0) (27.4) (30.9) (20.6)	51 (10.9) 91 (19.5) 160 (34.3) 133 (28.5) 31 (6.7)	(6.3) (14.8) (28.6) (30.8) (19.6)
Sex Female Male	243 (48.5) 258 (51.5)	(47.7) (52.3)	227 (48.7) 239 (51.3)	(47.9) (52.1)
Education level High school or less Postsecondary	121 (24.2) 379 (75.6)	(37.6) (62.3)	110 (23.6) 355 (76.2)	(36.8) (63.2)
Health literacy level Adequate Marginal/Inadequate	390 (77.8) 111 (22.2)	(78.8) (21.2)	367 (78.8) 99 (21.2)	(78.7) (21.3)
Cardiovascular health condition At risk of CVD With CVD	398 (79.4) 103 (20.6)	(81.9) (18.1)	374 (80.3) 92 (19.7)	(82.3) (17.7)
General health Excellent/Very good Good Fair/Poor	157 (31.3) 211 (42.1) 131 (26.1)	(33.0) (43.5) (23.3)	147 (31.5) 200 (42.9) 119 (25.5)	(33.7) (43.2) (23.1)
Multimorbidity^‡^ No comorbidity 2–3 chronic conditions 4–5 chronic conditions > 5 chronic conditions Comorbid mental and physical	51 (10.2) 208 (41.5) 151 (30.1) 91 (18.2) 176 (35.1)	(10.8) (39.3) (30.9) (19.0) (29.9)	49 (10.5) 197 (42.3) 137 (29.4) 83 (17.8) 160 (34.3)	(11.4) (39.7) (29.3) (19.2) (29.3)
Questionnaire completion time in minutes, mean (median; IQR)	25.7 (14.4; 11.1–19.1)		26.2 (14.5; 11.2–19.1)	
Single Ease Question score, median (IQR)	4 (3–5)		4 (3–5)	

*Abbreviations: CVD* cardiovascular disease, *IQR* interquartile range

^†^Thirty-four respondents that had selected the dominated alternative in the example choice set and one respondent that had missing data about their general health were excluded in the final model

^‡^Multimorbidity was assessed by asking respondents if they had ever been diagnosed by a health professional with one of 14 long-term health conditions, seven of which referred to a cardiovascular disease (e.g., angina, peripheral vascular/artery disease, heart failure) or risk factor (e.g., hypertension, diabetes outside of pregnancy). See Appendix A1 in Additional file 1 (pp. 13–16) for the list of conditions covered

### Preference estimates

In the main effects-only model (G-ROL1), all preference coefficients were statistically significant (*p* ≤ 0.001) and in the expected direction. Best attribute levels had positive coefficients indicating that their presence increased the probability of choosing a particular primary care practice, while worst attribute levels had negative coefficients indicating decreased choice probability. When the interaction between 24-to- 48-h accessibility and continuity of care was added to the model (G-ROL2), best continuity no longer had an independent effect on choice of primary care practice (*p* = 0.160). However, two out of the four interaction coefficients were statistically significant: best continuity had a positive impact on clinic choice when combined with moderate or best short-term accessibility. G-ROL1 and G-ROL2 models are presented in Appendix A5 (see Additional file 1). The final model (G-ROL3, Table 3) had the same statistically significant main and interaction effects as G-ROL2, as well as two additional statistically significant covariable interactions: best continuity of care had a greater effect on respondents aged 65 years or more (*p* < 0.001), and on those reporting having fair or poor general health (*p* = 0.008) compared to younger and healthier respondents, respectively. Unexplained heterogeneity was found for all but one main attribute level (providing ‘a lot’ of personalized information), as indicated by the size and statistical significance of the standard deviations of the main coefficients. This means that different respondents placed a different relative importance on most primary care attributes.

**Table 3:** Preference results of the final generalized rank-ordered logit model (G-ROL3)

Attributes and levels	Preference estimates				Marginal effects^†^, % (SD)
	**Coefficient**	**[95% CI]**	**SD**	**[95% CI of SD]**	
Listening to and respecting patient care preferences A little Moderate A lot	− 1.559^***^ Reference 0.248^***^	[− 1.829 to − 1.290] [0.106 to 0.390]	1.305^***^ 0.668^***^	[1.081 to 1.529] [0.472 to 0.864]	− 14.7 (7.5) Reference 2.6 (1.2)
Providing personalized information A little Moderate A lot	− 0.807^***^ Reference 0.292^***^	[− 0.942 to − 0.671] [0.176 to 0.408]	0.570^***^ 0.121	[0.333 to 0.807] [− 0.369 to 0.612]	− 7.6 (3.6) Reference 2.9 (1.2)
24-to- 48-h accessibility Rarely About every other time Always or almost	Reference 1.506^***^ 1.862^***^	[1.221 to 1.790] [1.477 to 2.247]	0.831^***^ 1.098^***^	[0.649 to 1.012] [0.852 to 1.344]	Reference 16.2 (8.5) 20.5 (8.1)
Continuity of care A little Moderate A lot	− 1.249^***^ Reference − 0.245	[− 1.634 to − 0.865] [− 0.526 to 0.037]	0.989^***^ 0.357^***^	[0.733 to 1.246] [0.144 to 0.570]	− 10.0 (5.2) Reference 5.1 (5.0)
Up-to-date clinical skills Every 8–10 years Every 4–5 years Every 1–2 years	− 1.528^***^ Reference 0.826^***^	[− 1.750 to − 1.307] [0.650 to 1.002]	1.191^***^ 0.991^***^	[0.987 to 1.396] [0.773 to 1.208]	− 14.1 (6.9) Reference 8.6 (3.5)
24-to- 48-h accessibility × Continuity of care About every other time × A little About every other time × A lot Always or almost × A little Always or almost × A lot	− 0.111 0.554^***^ 0.265 0.457^*^	[− 0.443 to 0.222] [0.218 to 0.889] [− 0.173 to 0.702] [0.102 to 0.812]			
Age × Continuity of care 65 years or older × A little 65 years or older × A lot	0.136 0.612^***^	[− 0.212 to 0.483] [0.345 to 0.878]			
General health × Continuity of care Fair or poor × A little Fair or poor × A lot	− 0.186 0.452^**^	[− 0.545 to 0.172] [0.120 to 0.784]			
τ	0.310^***^	[0.128 to 0.491]			
Log-pseudolikelihood	− 5106.282				
AIC	10,270.56				
BIC	10,501.14				
Observations	20,970				
Respondents	466				

*Abbreviations: AIC* Akaike information criterion, *BIC* Bayesian information criterion, *CI* confidence interval, *SD* standard deviation

^†^Interaction coefficients are used where applicable for calculating marginal effects but do not have their own marginal effect, as explained by Williams [65]

^***^p ≤.001

^**^p <.01

^*^p <.05

In the sensitivity analyses (see Appendix A6 in Additional file 1), removing the sampling weights made no difference on the sign or statistical significance of preference coefficients. However, one notable difference was observed when respondents selecting the dominated alternative in the example choice set were included in the model: the model’s correlation parameter (τ) increased from 0.310 to 0.495. Although sources of correlation cannot be disentangled from current choice models [66], this increase would be consistent with higher scale heterogeneity being introduced due to these participants responding more ‘randomly’ to the choice situations [62].

### Predicted choice

The rightmost column of Table 3 presents the marginal effects of attribute levels on predicted clinic choice according to the final model (G-ROL3). These marginal effects represent the average difference, in percentage points, in the probability of choosing a primary care practice with a given attribute level compared to a practice with the reference attribute level. We found that the average marginal effects associated with moving from the worst to the moderate level for all five attributes were, to varying degrees, of greater magnitude than the corresponding marginal effects associated with moving from the moderate to the best level. The attribute level with the largest impact on clinic choice was best 24-to- 48-h accessibility (+ 20.5% change in choice probability). However, contrary to other attributes, the worst rather than the moderate level was the reference for this attribute. By calculating the full range of predicted choice change attributable to each attribute across the included levels, up-to-date clinical skills was the most influential organizational feature (22.7% marginal effect range), followed by 24-to- 48-h accessibility (20.5%), listening to and respecting care preferences (17.3%), continuity of care (15.1%), and lastly, providing personalized information (10.5%). Improving short-term accessibility from worst to moderate level was the incremental change with the largest average marginal effect on predicted choice of a primary care practice (+ 16.2%).

Predicted choice probabilities for selected policy-relevant scenarios of primary care practices are presented in Table 4. The first three scenarios examined relate to primary care practices with the worst, best, and reference levels in all attributes. Their mean predicted choice probabilities were 3.1%, 83.8%, and 36.6%, respectively. The last three scenarios reflect incremental deviations from the reference setting. Improving continuity of care to the best level had only a minor effect on mean predicted choice probability (+ 2.2% change in choice probability compared to the reference scenario). Comparatively, improving accessibility to a moderate level had a much greater impact (+ 20.7%). However, this gain was almost nullified when the improvement in accessibility was achieved at the expense of the worst continuity (+ 2.9%).

**Table 4:** Choice probabilities of selected primary care setting scenarios

Attributes	Scenarios					
	**Worst**	**Best**	**Reference**	**Reference + best continuity**	**Reference + moderate accessibility**	**Reference + moderate accessibility + worst continuity**
Listening to and respecting patient care preferences	A little	A lot	Moderate	Moderate	Moderate	Moderate
Providing personalized information	A little	A lot	Moderate	Moderate	Moderate	Moderate
24-to- 48-h accessibility	Rarely	Always or almost	Rarely	Rarely	About every other time	About every other time
Continuity of care	A little	A lot	Moderate	A lot	Moderate	A little
Up-to-date clinical skills	Every 8–10 years	Every 1–2 years	Every 4–5 years	Every 4–5 years	Every 4–5 years	Every 4–5 years
Choice probability, % (SD)	3.1 (4.8)	83.8 (12.0)	36.6 (23.5)	38.8 (23.8)	57.3 (22.8)	39.5 (23.0)

*Abbreviations: SD* standard deviation

## Discussion

This study assessed patient preferences regarding key practice features for the organization of primary cardiovascular care by conducting a DCE study among a weighted sample of Quebec respondents with CVD or at CVD risk. All five attributes included in our study significantly influenced choices for primary care practices, but the organizational features that were most strongly valued by participants were keeping clinical skills up-to-date and rapid access to a professional in the event of a problem. Best continuity of care had no significant impact on patients when primary care practices had the worst short-term accessibility, but older patients and those in poorer general health valued best continuity more than others. Our respondents reported that completing the choice tasks was neither easy nor difficult. This is consistent with the balanced scenarios in our experimental design and the delicate trade-offs among key organizational attributes that were required of them, lending credibility to our results.

Our main findings resonate with other primary care DCE studies conducted in various jurisdictions. In England, Cheraghi-Sohi et al. [67] found that technical quality of care was among the attributes that primary care patients valued most regardless of clinical scenario, and that, in an urgent physical scenario, patients were willing to pay the most for a short waiting time. In their systematic review, Lim et al. [21] also reported that the attending professional’s experience or expertise showed robust evidence of influence on patients in the context of chronic disease care. However, due to the small number of chronic disease-specific DCE studies, the evidence was lacking for a number of attributes that are potentially important to patients. Our study contributes to strengthening the previously limited support for professional consideration of the patient’s perspective in the context of chronic disease [21]. Numerous studies have found that older patients tend to place greater emphasis on continuity of care [68]. Rubin et al. [69] reported that older patients placed a higher value on being able to see their own doctor rather than any available doctor for a primary care appointment. In Sweden, Hjelmgren and Anell [70] also found that older individuals and those in poor health expressed a stronger preference for registering with a specific doctor rather than a primary care team, emphasizing the importance of relational continuity in their healthcare. Moreover, the results of a recent study analyzing over 10 million consultations in 381 English primary care practices over a period of 11 years suggest that older patients, patients with multiple chronic conditions, and patients with mental health conditions are those for whom continuity of care may provide the highest productivity benefits [71].

However, providing rapid access to a clinic professional in the event of a problem had more influence than continuity of care in our study, a finding which seems to contrast with other DCE studies [69, 72, 73]. For example, Rubin et al. [69] concluded that speed of access was outweighed by continuity of care because their respondents were willing to wait a few extra days for an appointment with a doctor of their choice. In these studies, continuity of care was modelled dichotomously (e.g., doctor knows you well vs. does not know you), which likely affected the results and their interpretation. We observed that attaining the best level in the organizational attributes studied had less impact on choice than failing to achieve a moderate level. Thus, reports that a modest improvement in waiting time is not worth trading-off relational continuity would be consistent with our own results. A recent Canadian DCE which, similarly to our study, modelled wait time until appointment and familiarity with the provider as three-level attributes also found that timely access had more influence than continuity on patient choices for appointment bookings across a number of clinical scenarios [74].

To our knowledge, our DCE study was the first to specifically investigate the interactions between short-term accessibility and continuity of care. This enabled a finer understanding of how patients trade-off these two core and much-debated organizational features of primary care [6, 10, 14, 43–48]. Our results suggest that the value placed on short-term accessibility and continuity is, to some extent, conditional on each other. Respondents derived additional utility from best versus moderate continuity of care only when short-term accessibility to a professional in the primary care practice was at moderate or best level, and the combination of enhanced short-term access and best continuity produced a synergistic effect stronger than the sum of their individual main effects. Moreover, enhanced short-term accessibility in a context where continuity of care is at its worst resulted in only a trivial increase in choice probability compared to the reference scenario.

### Strengths and limitations

This study had many strengths. Our DCE study followed a blocked best-best design which maximized statistical efficiency while keeping respondent burden reasonable [42, 50, 75]. The attributes included were based on previous research that had used qualitative and quantitative data to identify the most important organizational features to optimally organize primary cardiovascular care for patients, from the perspectives of patients themselves as well as primary care providers [29]. All of the organizational features studied are amenable to policy or practice-level change. Even clinical skills, which may seem like an individual attribute of clinicians, can be supported by practice features such as a shared vision from clinic leaders, the practice's organizational structure (including the sharing of administrative resources, the presence of competence-maintenance mechanisms such as continuous professional development activities and chart audits, and the number of diagnostic and therapeutic procedures available through on-site technical facilities), as well as team processes that foster innovation and task orientation [13, 76, 77]. Our respondents came from various age groups, socioeconomic backgrounds and health status, reflecting the heterogeneous populations followed in primary care. The choice context in our survey was based on participants’ current clinical situation, which maximized the realism of the experiment. Our analyses were weighted to mitigate the overrepresentation of younger and more educated individuals that is typical of online panels [78], and accounted for both explained (by observable characteristics) and unexplained heterogeneity to minimize bias [24]. A vast array of relevant covariables were considered to explain heterogeneity in patient preferences on top of the usual sociodemographic characteristics. However, only two covariable interactions remained significant in our final model. According to Fiebig et al. [79], most group-specific effects are often rendered insignificant with the addition of random individual-specific effects in choice models, as done with our G-ROL models. Only a small proportion of respondents failed the dominance test in the example choice set, and our findings were robust to their inclusion as well as the removal of sampling weights.

However, our study also had some limitations. Although similarities were found with primary care DCE studies conducted in other countries, our results were based on respondents from a single jurisdiction and may not be generalizable to other settings. Furthermore, participants were self-selected and their eligibility was based on self-report. We cannot exclude that some people may have misrepresented themselves, although we partnered with a reputable survey firm that is regularly solicited by major private and public organizations including governments and universities. Internet use is ubiquitous in the adult Quebec population [80], but our data collection method may have excluded some of the most vulnerable cardiovascular patients lacking a minimum level of digital literacy or access to an Internet-connected device. Respondents who opened the invitation and completed the questionnaire first may also have been unrepresentative of our target population in ways that were not corrected by data weighting (e.g., personality biases [81]). As such, our preference estimates may not reflect true population values.

### Implications and future research

Based on the findings of this study, it is suggested that primary care practices should aim for a moderate level of attainment across all key organizational features covered, rather than focusing solely on achieving excellence in any single attribute. This balanced approach is deemed to be more advantageous. Thus, all other things being equal, the most impactful step that typical Quebec primary care practices should take to provide cardiovascular care that better aligns with patient preferences in this study would be to moderately improve their short-term accessibility. Amidst the debates between accessibility and continuity of care, primary care stakeholders should take note that the best continuity may appeal more to older and more frail patients, but all patients—including them—place significant importance on short-term accessibility in the event of a problem. Therefore, primary care practices that strive to provide excellent continuity of care to all patients without attending to their lack of short-term accessibility may be wasting much of their effort. Similarly, clinical settings that focus mainly on walk-in consultations and offer only minimal relational and informational continuity of care may not represent an attractive alternative for patients with or at risk of CVD. Finally, poor listening and respect for patient care preferences and longer clinical skill update cycles should especially be avoided, as these characteristics were powerful deterrents to our respondents.

We do not think that our findings are entirely due to the Quebec context because similar results have been observed elsewhere [21, 67, 68, 74]. However, this warrants further exploration in jurisdictions without significant primary care access issues. Our findings appear transferable to the management of other prevalent chronic physical health problems, many of which share common risk factors and follow-up requirements with CVD. However, this could also be examined more precisely in future studies. The preferences for primary care organization of younger patients, patients without any chronic condition, with only mental health issues or with complex socio-medical problems may also differ from those of adults followed for a chronic physical health problem. Finally, further methodological studies are required to explore whether the significant unexplained heterogeneity found in this and other DCE studies [24] may be reduced by incorporating additional observable characteristics or latent factors which were not accounted for in our analyses.

## Conclusions

Primary care is inherently multidimensional and complex, and decision-makers at both clinical and political levels often manage trade-offs between competing dimensions of care with little knowledge of the importance patients place on them. In this study, a weighted online sample of cardiovascular patients in Quebec significantly valued up-to-date clinical skills, 24-to- 48-h accessibility to a professional in the event of a problem, listening to and respecting patient care preferences, continuity of care, and provision of personalized information in choosing a primary care practice. Patients were more negatively influenced by worst levels in these key organizational features than they were positively attracted by best levels of achievement, suggesting that a balanced approach may be preferable to trying to shine in any single attribute. Keeping professionals’ clinical skills up to date at shorter intervals was the most important attribute overall, but improving short-term accessibility from worst to moderate level was the most influential incremental change. This was the first DCE study to examine and report significant interaction effects between short-term accessibility and continuity of primary care in the context of chronic disease. Best continuity was more valued by older patients and those in poorer general health, but had nonsignificant impact when short-term accessibility to a practice professional was at its worst. Improving accessibility at the expense of worse continuity of care also had negligible net effect. These findings underline the importance of joint monitoring of the potential impact on short-term accessibility and continuity of care of any reorganization of primary care services, to ensure that the changes result in significant net added value for patients with chronic diseases. The extent to which these preferences are aligned with population parameters or generalizable to other jurisdictions and subsets of primary care patients should be explored further.

## Supplementary Information

Supplementary Material 1: Appendix (Appendix A1 to Appendix A6)

## Data Availability

The datasets generated and analyzed during the current study are available in the Figshare repository, https://doi.org/10.6084/m9.figshare.26264096.v1.

## Abbreviations

CCHS: Canadian Community Health Survey
CVD: Cardiovascular disease
DCE: Discrete choice experiment
G-MNL: Generalized multinomial logit
G-ROL: Generalized rank-ordered logit
LEO: Leger Opinion
ROL: Rank-ordered logit
SEQ: Single Ease Question
