# Supplementary material for: Patient preferences for key organizational features of primary cardiovascular care in Quebec: a discrete choice experiment
Source: BMC Prim Care. 2025 Apr 10;26:103. doi: 10.1186/s12875-025-02810-4 (PMC11983794; doi:10.1186/s12875-025-02810-4)
Supplement: Supplementary file 1 — Supplementary Material 1: Appendix (Appendix A1 to Appendix A6) [file 12875_2025_2810_MOESM1_ESM.docx]

**Supplementary Material – Appendix**

[**Appendix A1.** English version of the online survey](#AppendixA1)

[**Appendix A2.** Characteristics influencing patient preferences for the organization of primary cardiovascular care](#AppendixA2)

[**Appendix A3.** Sample size calculations](#AppendixA3)

[**Appendix A4.** Methods for including covariable interactions in the final choice model (G-ROL3)](#AppendixA4)

[**Appendix A5.** G-ROL1 and G-ROL2 models](#AppendixA5)

[**Appendix A6.** Sensitivity analyses](#AppendixA6)

**Appendix A1. English version of the online survey**

| Our research firm is known for the accuracy of its results, thanks in part to the quality of its respondents! A few quality control questions have been placed throughout this questionnaire to ensure that you stay attentive. If you answer too many quality control questions incorrectly, your responses to this survey will not be considered. |
| --- |

Would you prefer to complete the survey in English or French?

English / Anglais

Français / French

[SECTION 1]

**Purpose of the Study:** The purpose of this study is to learn about patient and public preferences regarding the organization of cardiovascular care in Quebec’s family medicine clinics. You will be asked to complete a series of questions on this topic, as well as questions about your demographic profile and health status.

Your participation is on a free and voluntary basis. It should take you approximately 20 minutes. The study will involve approximately 500 participants. Not wanting to participate will not affect your usual care.

There are no known risks to participating in this study. You may choose not to answer any questions that make you uncomfortable. Please feel free to contact us for help.

There are no personal benefits for your participation. However, your participation is important because it will help us know about the preferences of people like you regarding how care is organized.

By agreeing to participate in this study, you do not waive any of your rights and you do not absolve the researcher responsible for this study and the institution from their civil and professional liability.

Privacy and Confidentiality: The information collected is coded and will remain strictly confidential. It will not be possible to identify you in any presentation or publication. The data will be kept on the CHUM's secure server for 10 years after the end of the project. You may obtain the study’s general results by requesting them from the researcher in charge, at the end of the study.

If you have any questions or problems related to the research project, please contact the student-researcher at any time by email at the following address: c.del.grande@umontreal.ca.

Should you have any questions about your rights as a participant in this study, you can contact the CHUM's local commissioner for complaints and service quality at [phone number].

The CHUM’s research ethics committee has approved and will monitor the study.

By checking yes below, I confirm that I have read the information and agree to participate in the survey. I will then be directed to the survey.

If you check no, your participation ends and we thank you.

Yes, I agree to be directed to the survey

No, I choose not to participate in this study and this will end my participation.

**COMMITMENT OF THE PRINCIPAL INVESTIGATOR AT THE CHUM**

I, along with the research team, agree to abide by the terms of the study as explained above.

Thank you for agreeing to participate in this study.

The following questions will help us determine your eligibility and participant profiles.

[SECTION 2]

**What is your age group?**

18 – 34 years

35 – 44 years

45 – 54 years

55 – 64 years

65 – 74 years

75 years or older

[If ineligible]

Thank you for your interest in our study.

Unfortunately, only people 35 years of age and over who live in Quebec and who have or are at risk of having a cardiovascular health problem can participate.

**Do you currently live in Quebec (province)?**

Yes

No

[If ineligible]

Thank you for your interest in our study.

Unfortunately, only people 35 years of age and over who live in Quebec and who have or are at risk of having a cardiovascular health problem can participate.

**Please indicate the first 3 characters of your postal code.**

_ _ _

**Please indicate your sex:**

Note: As indicated by Statistics Canada, transgender, transsexual, and intersex Canadians should indicate the sex (male or female) with which they most associate themselves.

**Row:**

Male

Female

[RANDOM QUALITY CONTROL QUESTION #1]

[If answered incorrectly]

Oops! Be careful. You answered a quality control question incorrectly. Please be sure to read the rest of the questions carefully. Thank you!

The next questions aim to determine if you currently have or are at risk of developing a cardiovascular (heart and blood vessels) health problem.

**Do you have hypertension (high blood pressure)?**

Yes

No

**In the past year, have you taken any medicine for high blood pressure?**

Yes

No

**Do you have high blood cholesterol or lipids?**

Yes

No

**In the past year, have you taken any medicine for high blood cholesterol or lipids?**

Yes

No

**Do you have heart disease (angina, ischemia, heart attack or myocardial infarction, heart failure, etc.)?**

Yes

No

**Have you ever been diagnosed with heart disease (angina, ischemia, heart attack or myocardial infarction, heart failure, etc.)?**

Yes

No

**Do you suffer from the effects of a stroke?**

Yes

No

**Do you have diabetes (outside of pregnancy only)?**

Yes

No

**Other than during pregnancy, has a health professional ever told you that you have diabetes?**

Yes

No

[If ineligible]

Thank you for your interest in our study.

Unfortunately, only people 35 years of age and over who live in Quebec and who have or are at risk of having a cardiovascular health problem can participate.

[SECTION 3]

You are eligible to participate in the study.

Please read the following instructions to complete the next section of the survey.

**INSTRUCTIONS**

The main section of the questionnaire contains nine scenarios.

Each scenario presents three fictional (imaginary) examples of family practice clinics that are organized differently to provide cardiovascular care to their patients.

*Cardiovascular health care is about preventing, treating and monitoring your heart and blood vessel health problems.*

In each scenario, you will be asked to choose, among the three choices offered, the family medicine clinic where you would prefer to be treated. You will then be asked to indicate your second choice from the two remaining clinics.

**Please answer as if you had to make these choices in real life.**

Clinic organization will be described in terms of the following five aspects:

1. **Listening to and respecting patient care preferences** – How much professionals listen to you in order to respect your requests, choices, preferences and motivation in care decisions. [*A lot*; *Moderate*; or *A little*]
2. **Providing personalized information** – How much detailed information about your own health status (check-ups, personal risks, etc.) is shared by professionals during consultations. [*A lot*; *Moderate*; or *A little*]
3. **24-to-48-hour accessibility** – How often you can reach a clinic professional within 24-48 hours if you have a problem, either on site, by phone or by teleconsultation (e.g., video conferencing). [*Always or almost*; *About every other time*; or *Rarely*]
4. **Continuity of care** – How much the consulted professionals know you and have easy access to all your health information to ensure there is a link between your visits. [*A lot*; *Moderate*; or *A little*]
5. **Up-to-date clinical skills** – The speed with which the clinic's professionals keep up with new ways to better care for you. [*Every 1-2 years*; *Every 4-5 years*; or *Every 8-10 years*]

**PLEASE NOTE:** Although the fictitious clinics are always identified by the letters A, B and C, their characteristics change from one scenario to the next, so please consider them carefully.

Here is an easier example of a situation to familiarize yourself with the task. Please complete it before continuing.

At any time, you can hover or click on an aspect of care to bring up the more detailed description.

**Thank you in advance for providing answers that truly reflect your opinion.**

If you were to receive cardiovascular care at one of the following family practice clinics:

|  | **Clinic A** | **Clinic B** | **Clinic C** |
| --- | --- | --- | --- |
| **Listening to and respecting patient care preferences** | A little | A lot | Moderate |
| **Providing personalized information** | A little | Moderate | Moderate |
| **24-to-48-hour accessibility** | Rarely | Always or almost | About every other time |
| **Continuity of care** | A little | A lot | Moderate |
| **Up-to-date clinical skills** | Every 8-10 years | Every 1-2 years | Every 4-5 years |

| **Which one would you choose?** *(first choice)* |  |  |  |
| --- | --- | --- | --- |
| **Which of the remaining two clinics would you choose?** *(second choice)* |  |  |  |

Perfect!

We are now going to present the nine main scenarios of the survey.

Afterwards, we will ask you some additional questions to better understand your answers.

[Random allocation to block A or B]

[SCENARIO #1 of 9 → to SCENARIO #4 of 9 of assigned block]

[RANDOM QUALITY CONTROL QUESTION #2]

[If answered incorrectly 1/2]

Oops! Be careful. You answered a quality control question incorrectly. Please be sure to read the rest of the questions carefully. Thank you!

[If answered incorrectly 2/2]

Unfortunately, you answered too many quality control questions incorrectly. Your responses to this survey will not be considered and you will not earn any LEO points or chances. [END OF SURVEY]

[SCENARIO #5 of 9 → to SCENARIO #9 of 9 of assigned block]

Thank you for completing all the scenarios.

**Overall, how difficult or easy was it to make your choices?**

| *Very difficult* | |  | | | *Very easy* | |
| --- | --- | --- | --- | --- | --- | --- |
| **1** | **2** | **3** | **4** | **5** | **6** | **7** |

[SECTION 4]

The following questions will help us to better understand the organizational preferences that others like you may have. Please remember that all of your responses remain anonymous.

**Which category best describes your current main occupation?**

*If you have more than one occupation, select the one that currently takes up most of your time.*

Working at a paid job or business / self-employed

Looking for paid work

Going to school

Household work / Caring for children

Retired

Maternity or paternity leave

Long term illness

Volunteering

Other

Do not wish to answer

**What is your level of education?**

*Select one response based on your highest completed level of education.*

Incomplete primary studies

Primary

High school (secondary)

College or professional/vocational

University

Do not wish to answer

**To the best of your knowledge, which category applies to the total income of all members of your household, before taxes and other deductions, during the past year?**

$5,000 to less than $10,000

$10,000 to less than $15,000

$15,000 to less than $20,000

$20,000 to less than $30,000

$30,000 to less than $40,000

$40,000 to less than $50,000

$50,000 to less than $60,000

$60,000 to less than $70,000

$70,000 to less than $80,000

$80,000 to less than $90,000

$90,000 to less than $100,000

$100,000 to less than $150,000

$150,000 or more

I don't know

Do not wish to answer

**How important is health to you?**

*Extremely* important

*Very* important

*Moderately* important

*Slightly* important

*Not at all* important

Do not wish to answer

**In general, would you say your health (including your physical, mental and social well-being) is:**

Excellent

Very good

Good

Fair

Poor

I don't know

Do not wish to answer

[RANDOM QUALITY CONTROL QUESTION #3]

[If answered incorrectly 1/2]

Oops! Be careful. You answered a quality control question incorrectly. Please be sure to read the rest of the questions carefully. Thank you!

[If answered incorrectly 2/2]

Unfortunately, you answered too many quality control questions incorrectly. Your responses to this survey will not be considered and you will not earn any LEO points or chances. [END OF SURVEY]

**Have you ever been diagnosed with one of the following long-term health conditions by a health professional?**

If so, please estimate the current severity of the problem in your case.

**Hypertension (high blood pressure)**

No

Yes – Severity: Mild

Yes – Severity: Moderate

Yes – Severity: Severe

I don't know / I don't want to answer

**High cholesterol or blood fats**

No

Yes – Severity: Mild

Yes – Severity: Moderate

Yes – Severity: Severe

I don't know / I don't want to answer

**Angina, ischemia, or heart attack (myocardial infarction)**

No

Yes – Severity: Mild

Yes – Severity: Moderate

Yes – Severity: Severe

I don't know / I don't want to answer

**Cerebrovascular accident (stroke), transient ischemic attack, cerebrovascular thrombosis**

No

Yes – Severity: Mild

Yes – Severity: Moderate

Yes – Severity: Severe

I don't know / I don't want to answer

**Peripheral vascular/artery disease (blood flow problem in your legs)**

No

Yes – Severity: Mild

Yes – Severity: Moderate

Yes – Severity: Severe

I don't know / I don't want to answer

**Heart failure**

No

Yes – Severity: Mild

Yes – Severity: Moderate

Yes – Severity: Severe

I don't know / I don't want to answer

**Diabetes (outside of pregnancy only)**

No

Yes – Severity: Mild

Yes – Severity: Moderate

Yes – Severity: Severe

I don't know / I don't want to answer

**Asthma**

No

Yes – Severity: Mild

Yes – Severity: Moderate

Yes – Severity: Severe

I don't know / I don't want to answer

**Chronic bronchitis, emphysema or chronic obstructive pulmonary disease (COPD)**

No

Yes – Severity: Mild

Yes – Severity: Moderate

Yes – Severity: Severe

I don't know / I don't want to answer

**Arthritis (osteoarthritis, rheumatoid arthritis, gout, etc.)**

No

Yes – Severity: Mild

Yes – Severity: Moderate

Yes – Severity: Severe

I don't know / I don't want to answer

**Cancer**

No

Yes – Severity: Mild

Yes – Severity: Moderate

Yes – Severity: Severe

I don't know / I don't want to answer

**Bowel disease (Crohn’s disease, ulcerative colitis)**

No

Yes – Severity: Mild

Yes – Severity: Moderate

Yes – Severity: Severe

I don't know / I don't want to answer

**Mood disorder (depression, bipolar disorder, mania or dysthymia)**

No

Yes – Severity: Mild

Yes – Severity: Moderate

Yes – Severity: Severe

I don't know / I don't want to answer

**Anxiety disorder (phobia, obsessive-compulsive disorder, panic disorder)**

No

Yes – Severity: Mild

Yes – Severity: Moderate

Yes – Severity: Severe

I don't know / I don't want to answer

**How often do you have someone (family, friends, neighbors, doctors, pharmacists, etc.) help you read materials from your medical clinic or the hospital?**

Always

Often

Sometimes

Occasionally

Never

**How often do you have problems learning about your health condition because of difficulty understanding written information?**

Always

Often

Sometimes

Occasionally

Never

**How often do you have a problem understanding what a health professional is telling you about your health condition?**

Always

Often

Sometimes

Occasionally

Never

**How confident are you filling out medical forms by yourself?**

Not at all

A little bit

Somewhat

Quite a bit

Extremely

Please use the ruler below to answer the following final questions.

These focus on your readiness to change your lifestyle to improve your health.

If you are definitely not ready to change, you should indicate ‘1’. If you are already trying hard to change, you should indicate ‘10’.

| Not ready to change |  |  | Unsure |  |  | Ready to change |  |  | Trying to change |
| --- | --- | --- | --- | --- | --- | --- | --- | --- | --- |
| **1** | **2** | **3** | **4** | **5** | **6** | **7** | **8** | **9** | **10** |

**How ready are you to change your diet (what and how much you eat) to improve your health?**

| Not ready to change |  |  | Unsure |  |  | Ready to change |  |  | Trying to change |
| --- | --- | --- | --- | --- | --- | --- | --- | --- | --- |
| **1** | **2** | **3** | **4** | **5** | **6** | **7** | **8** | **9** | **10** |

**How ready are you to change your level of physical activity (the activities you do, their frequency, intensity or duration) to improve your health?**

| Not ready to change |  |  | Unsure |  |  | Ready to change |  |  | Trying to change |
| --- | --- | --- | --- | --- | --- | --- | --- | --- | --- |
| **1** | **2** | **3** | **4** | **5** | **6** | **7** | **8** | **9** | **10** |

**How ready are you to change your tobacco consumption to improve your health?**

*If you don't smoke, please indicate 'I don't smoke'.*

| Not ready to change |  |  | Unsure |  |  | Ready to change |  |  | Trying to change |
| --- | --- | --- | --- | --- | --- | --- | --- | --- | --- |
| **1** | **2** | **3** | **4** | **5** | **6** | **7** | **8** | **9** | **10** |

I don’t smoke

**How ready are you to change your alcohol consumption to improve your health?**

*If you don't drink alcohol, please indicate 'I don't drink'.*

| Not ready to change |  |  | Unsure |  |  | Ready to change |  |  | Trying to change |
| --- | --- | --- | --- | --- | --- | --- | --- | --- | --- |
| **1** | **2** | **3** | **4** | **5** | **6** | **7** | **8** | **9** | **10** |

I don’t drink

Thank you for taking the time to complete our survey!

**Thanks to your responses, we will be able to share with family medicine clinics in Quebec which aspects of cardiovascular care organization are most important.**

[END OF SURVEY]

**Appendix A2. Characteristics influencing patient preferences for the organization of primary cardiovascular care**

While conducting a modified Delphi study that aimed to identify the top priorities shared by primary care patients and clinicians in Quebec (Canada) for the organization of cardiovascular care in primary care settings [1], we asked the Delphi panelists during the first survey round (n=36, 20 patients and 16 clinicians) to formulate, in free-text fields, up to five patient characteristics that may influence patient preferences regarding these priorities. Their responses were summarized into mutually exclusive items.

In the second round, the Delphi panelists (n=33, 17 patients and 16 clinicians) were asked: “*To what extent do you think the following characteristics may influence patient preferences for the organization of their primary cardiovascular care?*” The response scale included three categories (‘*a little*’, ‘*moderately*’, ‘*a lot*’), with a ‘*don’t know*’ option. To avoid order effects, the characteristics were presented to each respondent in random order.

On top of typical sociodemographic characteristics (such as age, sex, socioeconomic status, or main occupation), we intended to include five additional patient characteristics in our discrete choice experiment survey. This number was predetermined to keep survey length short. The five additional characteristics were selected based on the proportion of panelists indicating that they may have ‘*a lot*’ of influence on patient preferences.

During Round 1, Delphi panelists elicited 19 unique patient characteristics. They are presented below, in descending order based on the proportion of panelists indicating, during Round 2, that they may have ‘*a lot*’ of influence on patient preferences for the organization of primary cardiovascular care.

**Table A2-1.** Patient characteristics influencing patient preferences for the organization of primary cardiovascular care

| **Patient characteristics** | **Number of panelists rating ‘a lot’ of influence on patient preferences (%**^†^**)** | **Included in DCE survey?** |
| --- | --- | --- |
| Number and type of chronic health problems (comorbidities) | 29 (90.6) | YES |
| Readiness to make lifestyle changes | 25 (78.1) | YES |
| Health literacy level | 23 (71.9) | YES |
| Severity of the cardiovascular health condition | 23 (71.9) | YES |
| Importance attached to health | 22 (68.8) | YES |
| Functional capacity (ability to perform daily tasks without difficulty) | 21 (65.6) | NO |
| Ease of getting around to get to appointments (mobility) | 21 (65.6) | NO |
| Health-related anxiety or distress | 20 (62.5) | NO |
| Socioeconomic status | 19 (59.4) | YES^‡^ |
| Availability or flexibility level in the schedule | 18 (56.3) | NO |
| Age | 17 (53.1) | YES^‡^ |
| Requiring an accompanying person for appointments | 16 (50.0) | NO |
| Social isolation level | 15 (46.9) | NO |
| Living in a remote area | 14 (43.8) | NO |
| Household composition | 14 (43.8) | NO |
| Living in a deprived neighborhood | 12 (37.5) | NO |
| Main occupation | 11 (34.4) | YES^‡^ |
| Language spoken at home | 10 (31.3) | NO |
| Ethnicity, culture, religion | 5 (15.6) | NO |

DCE indicates discrete choice experiment.

^†^Percentages were calculated based on 32 valid responses received.

^‡^These patient characteristics were included in the DCE survey because of their typicality in subgroup analyses. Sex was also included in the DCE survey although none of the Delphi panelists had mentioned it as a relevant characteristic during Round 1. Socioeconomic status was accounted for in the DCE survey by measuring the level of education and household income.

REFERENCE

1. Del Grande C, Kaczorowski J, Pomey M-P. What are the top priorities of patients and clinicians for the organization of primary cardiovascular care in Quebec? A modified e-Delphi study. PLoS One. 2023;18:e0280051.

**Appendix A3. Sample size calculations**

According to Johnson and Orme’s rule of thumb [1], the minimum sample size (N) required for estimating main effects can be estimated according to the following equation:

**N** ≥ 500*c* ÷ (*t* × *a*)

where *c* is the number of analysis cells, which equals to the largest number of levels for any one attribute; *t* is the number of choice tasks; and *a* is the number of alternatives per choice task.

In our study, this translated to: **N** ≥ 500 × 3 ÷ (9 × 3) = 55.6 ≈ **56**

When considering all two-way interactions, *c* is equal to the largest product of levels of any two attributes.

In our study, this translated to: **N** ≥ 500 × 3×3 ÷ (9 × 3) = 166.7 ≈ **167**

REFERENCE

1. Orme BK. Getting Started with Conjoint Analysis: Strategies for Product Design and Pricing Research. 2nd ed. Madison, WI: Research Publishers, LLC; 2010.

**Appendix A4.** **Methods for including covariable interactions in the final choice model (G-ROL3)**

All covariables assessed in the second and last survey sections of the discrete choice experiment survey (see [Appendix A1](#AppendixA1)) were interacted with each attribute of the discrete choice experiment. Main occupation was removed due to presence of multicollinearity with age. We also explored preference heterogeneity between respondents having established CVD vs. being only at CVD risk.

The choice of parameters for each covariable tested is listed below.

**Table A4-1. Parameters for the covariables interacted with main DCE attributes**

| **Covariables** | **Parameters** |
| --- | --- |
| Age | [0] Less than 65 years old  [1] 65 years or older |
| Sex | [0] Male  [1] Female |
| CVD status | [0] Only at CVD risk  [1] With CVD |
| Main occupation^†^ | [0] Not retired  [1] Retired |
| Level of education | [0] Postsecondary  [1] High school or less |
| Household income | [0] $40,000 or more  [1] Less than $40,000 |
| General health | [0] Excellent, very good or good  [1] Fair or poor |
| Number of chronic comorbidities | Linear (minimum – maximum) |
| Type of chronic comorbidities | [0] No comorbidity or comorbid physical only  [1] Comorbid mental and physical |
| Severity of the cardiovascular health condition | [0] Mild or moderate cardiovascular health condition  [1] Severe cardiovascular health condition |
| Importance attached to health | [0] Very, moderately, slightly or not at all important  [1] Extremely important |
| Health literacy level | [0] Adequate (scores between 17 and 20)  [1] Inadequate or marginal (scores between 4 and 16) |
| Readiness to make lifestyle changes | [0] Unsure, ready to change or trying to change lifestyle (score between 4 and 10 on at least one readiness ruler)  [1] Not ready to make any lifestyle change (scores between 1 and 3 on all readiness rulers) |

CVD indicates cardiovascular disease; DCE, discrete choice experiment.

^†^Removed due to multicollinearity with age.

To reduce computation times, standard rank-ordered logit (ROL) models estimated using Stata’s built-in cmrologit command [1] were used to identify the covariable interactions to include in generalized rank-ordered logit (G-ROL) modeling.

All statistically significant interactions (p < 0.05) found in standard ROL modeling were added to the G-ROL2 model. Nonsignificant covariables in the expanded G-ROL model were removed in a stepwise fashion until only statistically significant interactions remained in the final G-ROL3 model.

REFERENCE

1. StataCorp. Stata Choice Models Reference Manual: Release 17. Stata Press; 2021. <https://www.stata.com/manuals/cm.pdf>

**Appendix A5. G-ROL1 and G-ROL2 models**

**Table A5-1.** Preference estimates for the G-ROL1 and G-ROL2 models.

| **Attributes and levels** | **Coefficients [95% CI]** | |
| --- | --- | --- |
|  | **G-ROL1** | **G-ROL2** |
| Listening to and respecting patient care preferences  A little  *SD of A little*  Moderate^†^  A lot  *SD of A lot* | -1.493*** [-1.753 to -1.233]  *1.253*** [1.045 to 1.461]*  .  0.231*** [0.099 to 0.362]  *0.676*** [0.479 to 0.873]* | -1.530*** [-1.787 to -1.273]  *1.242*** [1.048 to 1.436]*  .  0.239** [0.087 to 0.391]  *0.702*** [0.491 to 0.913]* |
| Providing personalized information  A little  *SD of A little*  Moderate^†^  A lot  *SD of A lot* | -0.754*** [-0.871 to -0.636]  *0.535*** [0.293 to 0.777]*  .  0.290*** [0.171 to 0.409]  *0.247* [0.032 to 0.463]* | -0.799*** [-0.935 to -0.662]  *0.524*** [0.208 to 0.840]*  .  0.284*** [0.171 to 0.397]  *0.203 [-0.045 to 0.452]* |
| 24-to-48-hour accessibility  Rarely^†^  About every other time  *SD of About every other time*  Always or almost  *SD of Always of almost* | .  1.618*** [1.337 to 1.900]  *0.871*** [0.603 to 1.140]*  2.063*** [1.672 to 2.453]  *1.151*** [0.805 to 1.496]* | .  1.500*** [1.186 to 1.813]  *0.781*** [0.562 to 1.000]*  1.816*** [1.458 to 2.173]  *1.083*** [0.836 to 1.330]* |
| Continuity of care  A little  *SD of A little*  Moderate^†^  A lot  *SD of A lot* | -1.253*** [-1.460 to -1.046]  *0.990*** [0.708 to 1.272]*  .  0.498*** [0.355 to 0.641]  *0.310 [-0.145 to 0.766]* | -1.246*** [-1.558 to -0.934]  *0.956*** [0.735 to 1.178]*  .  0.181 [-0.071 to 0.434]  *0.326 [-0.010 to 0.662]* |
| Up-to-date clinical skills  Every 8-10 years  *SD of Every 8-10 years*  Every 4-5 years^†^  Every 1-2 years  *SD of Every 1-2 years* | -1.466*** [-1.671 to -1.261]  *1.250*** [0.962 to 1.539]*  .  0.784*** [0.612 to 0.957]  *0.925*** [0.684 to 1.166]* | -1.541*** [-1.746 to -1.336]  *1.190*** [0.975 to 1.404]*  .  0.813*** [0.640 to 0.986]  *0.987*** [0.738 to 1.237]* |
| 24-to-48-hour accessibility × Continuity of care  About every other time × A little  About every other time × A lot  Always or almost × A little  Always or almost × A lot |  | -0.092 [-0.461 to 0.276]  0.531** [0.192 to 0.869]  0.290 [-0.191 to 0.772]  0.459* [0.093 to 0.824] |
| τ | 0.332*** [0.136 to 0.528] | 0.354*** [0.209 to 0.500] |
| Log-pseudolikelihood | -5154.060 | -5139.133 |
| AIC | 10350.12 | 10328.27 |
| BIC | 10517.13 | 10527.09 |
| Observations | 21015 | 21015 |
| Respondents | 467 | 467 |

AIC indicates Akaike information criterion; BIC, Bayesian information criterion; CI, confidence interval; G-ROL, generalized rank-ordered logit; SD, standard deviation.

^†^Reference level.

***p ≤ .001.

**p < .01.

*p < .05.

**Appendix A6. Sensitivity analyses**

**Table A6-1.** Preference estimates for the sensitivity analyses

| **Attributes and levels** | **Coefficients [95% CI]** | |
| --- | --- | --- |
|  | **G-ROL3, with unweighted data** | **G-ROL3, including respondents (n=33^†^) who had selected the dominated alternative in the example choice set** |
| Listening to and respecting patient care preferences  A little  *SD of A little*  Moderate^‡^  A lot  *SD of A lot* | -1.528*** [-1.745 to -1.311]  *1.303*** [1.109 to 1.498]*  .  0.257*** [0.137 to 0.376]  *0.683*** [0.493 to 0.874]* | -1.356*** [-1.597 to -1.115]  *1.284*** [1.039 to 1.528]*  .  0.308*** [0.144 to 0.472]  *0.709*** [0.365 to 1.053]* |
| Providing personalized information  A little  *SD of A little*  Moderate^‡^  A lot  *SD of A lot* | -0.754*** [-0.882 to -0.626]  *0.663*** [0.488 to 0.839]*  .  0.301*** [0.205 to 0.398]  *0.252 [-0.088 to 0.592]* | -0.759*** [-0.902 to -0.616]  *0.525*** [0.308 to 0.741]*  .  0.284*** [0.172 to 0.395]  *0.212 [-0.054 to 0.477]* |
| 24-to-48-hour accessibility  Rarely^‡^  About every other time  *SD of About every other time*  Always or almost  *SD of Always of almost* | .  1.525*** [1.263 to 1.786]  *0.699*** [0.464 to 0.934]*  1.727*** [1.453 to 2.000]  *1.011*** [0.740 to 1.282]* | .  1.423*** [1.109 to 1.737]  *0.676*** [0.442 to 0.910]*  1.787*** [1.348 to 2.227]  *0.980*** [0.661 to 1.300]* |
| Continuity of care  A little  *SD of A little*  Moderate^‡^  A lot  *SD of A lot* | -1.312*** [-1.605 to -1.018]  *0.913*** [0.708 to 1.118]*  .  -0.179 [-0.417 to 0.058]  *0.428*** [0.167 to 0.689]* | -1.306*** [-1.698 to -0.913]  *0.970*** [0.681 to 1.260]*  .  -0.218 [-0.503 to 0.067]  *0.259 [-0.067 to 0.586]* |
| Up-to-date clinical skills  Every 8-10 years  *SD of Every 8-10 years*  Every 4-5 years^‡^  Every 1-2 years  *SD of Every 1-2 years* | -1.459*** [-1.651 to -1.267]  *1.183*** [1.010 to 1.355]*  .  0.755*** [0.615 to 0.896]  *0.983*** [0.807 to 1.159]* | -1.433*** [-1.672 to -1.193]  *1.185*** [0.939 to 1.430]*  .  0.741*** [0.567 to 0.915]  *0.952*** [0.699 to 1.205]* |
| 24-to-48-hour accessibility × Continuity of care  About every other time × A little  About every other time × A lot  Always or almost × A little  Always or almost × A lot | -0.137 [-0.415 to 0.142]  0.465*** [0.207 to 0.724]  0.236 [-0.97 to 0.569]  0.522*** [0.251 to 0.793] | -0.072 [-0.404 to 0.261]  0.476** [0.139 to 0.812]  0.319 [-0.111 to 0.749]  0.410* [0.053 to 0.767] |
| Age × Continuity of care  65 years or older × A little  65 years or older × A lot | 0.016 [-0.269 to 0.301]  0.417*** [0.171 to 0.663] | 0.132 [-0.237 to 0.502]  0.565*** [0.294 to 0.837] |
| General health × Continuity of care  Fair or poor × A little  Fair or poor × A lot | 0.013 [-0.289 to 0.314]  0.429** [0.153 to 0.704] | -0.119 [-0.498 to 0.259]  0.489** [0.154 to 0.824] |
| τ | 0.346*** [0.178 to 0.515] | 0.495*** [0.268 to 0.722] |
| Log-pseudolikelihood | -5186.228 | -5719.155 |
| AIC | 10430.46 | 11496.31 |
| BIC | 10661.03 | 11728.87 |
| Observations | 20970 | 22455 |
| Respondents | 466 | 499 |

AIC indicates Akaike information criterion; BIC, Bayesian information criterion; CI, confidence interval; G-ROL, generalized rank-ordered logit; SD, standard deviation.

^†^Thirty-four respondents had selected the dominated alternative in the example choice set, but one of them had missing data about their general health and was therefore excluded from the model.

^‡^Reference level.

***p ≤ .001.

**p < .01.

*p < .05.
